# Therapeutic potential of D-amino acid oxidase inhibitors for cognitive impairment associated with schizophrenia: learnings from luvadaxistat

**DOI:** 10.1093/ijnp/pyae066

**Published:** 2025-01-04

**Authors:** Ryan T Terry-Lorenzo, Reuben H Fan, Ni A Khin, Jaskaran B Singh

**Affiliations:** Neurocrine Biosciences, Inc., San Diego, CA, United States; Neurocrine Biosciences, Inc., San Diego, CA, United States; Neurocrine Biosciences, Inc., San Diego, CA, United States; Neurocrine Biosciences, Inc., San Diego, CA, United States

**Keywords:** schizophrenia, treatment, DAAO inhibitor, NBI-1065844, luvadaxistat

## Abstract

Hypofunction of the N-methyl-D-aspartate receptor (NMDAR) has been proposed to underlie the pathophysiology of schizophrenia, suggesting that promoting NMDAR activity may alleviate the negative or cognitive symptoms associated with schizophrenia. To circumvent excitotoxicity that may accompany direct agonism of the glutamate binding site on the NMDAR, therapeutic trials have focused on targeting the glycine binding site on the NMDAR. Direct administration of either glycine or D-serine, both of which are endogenous coagonists at the NMDAR glycine site, has yielded mixed outcomes across an array of clinical trials investigating different doses or patient populations. Furthermore, directly administering D-serine and glycine is challenging, and thus attention has turned to alternative, indirect methods that increase endogenous D-serine and glycine levels in the brain, such as D-amino acid oxidase (DAAO) inhibitors and glycine transporter 1 inhibitors, respectively. In this review, we provide an overview of the evidence supporting the potential of NMDAR modulators in general, and DAAO inhibitors in particular, as potential adjunctive treatments for schizophrenia. We also discuss the preclinical and clinical data related to luvadaxistat, an investigational highly selective and potent DAAO inhibitor that was under development for the treatment of the cognitive impairment associated with schizophrenia.

## THE NMDAR HYPOFUNCTION MODEL OF SCHIZOPHRENIA

**Figure 1.**
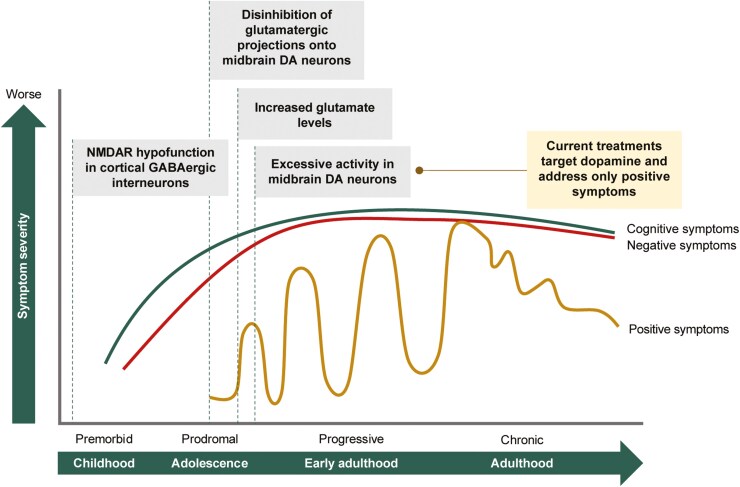
The course of schizophrenia symptoms over time and early changes in glutamate and dopamine systems thought to underlie the pathophysiology of schizophrenia. This figure was adapted from McCutcheon et al.^2^ Mosolov and Yaltonskaya,^3^ Sommer et al.,^4^ all with permission under the terms of the CC BY 4.0 license (https://creativecommons.org/licenses/by/4.0/). This figure was further modified with information from Murray et al.,^5^ Nakazawa et al.^6^ Abbreviations: DA, dopaminergic; GABAergic, gamma-aminobutyric acidergic; NMDAR, N-methyl-D-aspartate receptor.

Schizophrenia is a serious, chronic mental illness and is one of the leading causes of disability worldwide.^1^ Schizophrenia is characterized by positive (eg, hallucination, delusion, disorganized thoughts/behavior), negative (eg, alogia, anhedonia, affective flattening, asociality, avolition), and cognitive symptoms (eg, attention deficits, inability to concentrate, impaired short-term memory) (Figure 1).^2,7^ Most individuals with schizophrenia experience clinically relevant negative (60%) and cognitive symptoms (80%).^8,9^ Cognitive impairment associated with schizophrenia (CIAS) contributes to ongoing chronic disability in patients with schizophrenia and is associated with a greater degree of debilitating functional outcomes.^10-12^ However, approved pharmacological treatments predominantly target dopamine and exhibit greater effects on positive symptoms (Figure 1), leaving an unmet need for treatment options that counter the negative and cognitive symptoms of schizophrenia.^13^

The N-methyl-D-aspartate receptor (NMDAR) hypofunction model of schizophrenia proposes that deficits in NMDAR signaling underlie the pathophysiology of schizophrenia.^14^ Consistent with the NMDAR hypofunction hypothesis, the administration of NMDAR antagonists such as phencyclidine (PCP) and ketamine partially recapitulates the symptoms of schizophrenia in animals and humans.^15^ The discovery of anti-NMDAR encephalitis in 2007, an autoimmune disorder in which NMDAR autoantibodies cause internalization of NMDARs, and patients present with severe psychiatric and cognitive symptoms that are reminiscent of schizophrenia,^16-18^ has further supported the hypothesis linking NMDAR hypofunction and psychosis.

In addition to the above, genetic, biomarker, and postmortem evidence support a link between patients with schizophrenia and dysregulation of NMDARs. In human genome-wide association studies, variants of NMDA signaling genes and synaptic pathways have been identified as candidate risk genes for schizophrenia,^19^ with NMDAR genetic variants having been specifically linked to cognitive impairments in schizophrenia.^20^ In biomarker studies, NMDAR-dependent auditory processes such as mismatch negativity (MMN) and auditory steady-state response (ASSR) were shown to be impaired in patients with schizophrenia and associated with cognitive dysfunction.^21,22^ In a meta-analysis of postmortem studies, expression of specific NMDAR subunits in the prefrontal cortex was found to be significantly lower in patients with schizophrenia than in people without schizophrenia.^23^ Moreover, postmortem analysis also found that cortical dendritic spine density was reduced in patients with schizophrenia compared with people without schizophrenia, further implicating impairment in excitatory synapses in the pathophysiology of schizophrenia.^24^

## PROOF-OF-CONCEPT INTERVENTIONAL STUDIES DEMONSTRATE THAT PROMOTING NMDAR ACTIVITY AT THE GLYCINE SITE CAN TREAT SCHIZOPHRENIA

**Figure 2.**
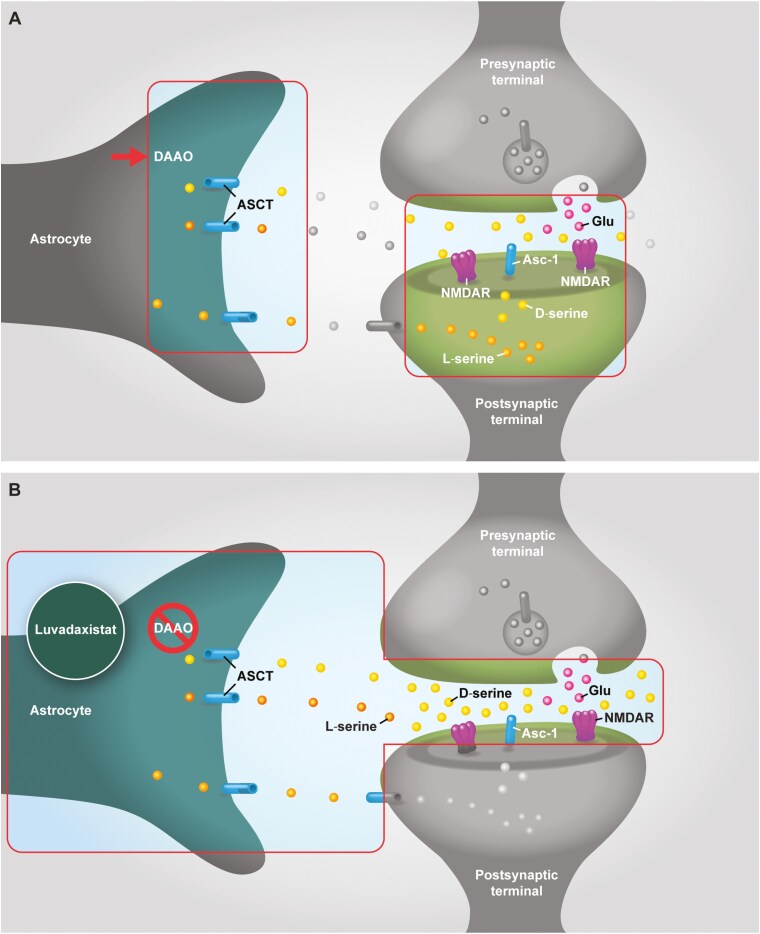
NMDA receptor hypofunction in the pathophysiology of schizophrenia. (A) After being synthesized from L‐serine by serine racemase, D‐serine is released into the synaptic cleft, allowing it to bind to the glycine modulatory sites on the synaptic NMDAR. The binding of both D-serine and glutamate to the NMDAR activates the receptor and elicits an action potential. D-serine reuptake is mediated by ASCT for its degradation by DAAO in astrocytes (B) Mechanism of action of DAAO inhibitors, a potential investigational treatment for cognitive impairment associated with schizophrenia. Inhibition of DAAO is thought to increase D-serine levels in the synapse. Increasing the levels of D-serine, a coagonist of the NMDARs, may reverse NMDA hypofunction. Abbreviations: Asc-1, alanine-serine-cysteine transporter 1; ASCT, alanine-serine-cysteine -threonine transporter; DAAO, D-amino acid oxidase; Glu, glutamate; NMDAR, N‐methyl‐D-aspartate receptor.

Given the multiple lines of evidence supporting the NMDAR hypofunction model described above, it has been theorized that promoting the activity of the NMDAR could alleviate schizophrenia symptoms. NMDARs are activated by the binding of coagonists at 2 distinct sites, defined by endogenous agonists: (1) the glutamate site and (2) the glycine site, which also binds and is activated by D-serine (Figure 2A).^25^ D-serine is synthesized from L-serine by serine racemase in neurons and, to a lesser degree, in astrocytes.^25,26^ A “shuttle” hypothesis proposes that neuronal D-serine is transported to astrocytes for vesicular storage by alanine-serine-cysteine transporters (ASCTs in astrocytes; Asc-1 in neurons)^26-29^; glycine transport can also be mediated by Asc-1.^29^ Once released into the synaptic cleft, D-serine binds to the glycine site on the synaptic NMDAR. Glutamate is also released from the presynaptic terminal into the synapse. Together, D-serine and glutamate bind to and activate the NMDAR, allowing positively charged ions to flow through the plasma membrane and elicit an action potential (Figure 2A).^25^ D-serine is also removed from the synaptic cleft via ASCT and Asc-1 ^27^.

Because direct agonism of NMDARs at the glutamate binding site may lead to excitotoxicity, the majority of therapeutic trials have focused on compounds targeting the glycine site, and several glycine site NMDAR modulators have shown efficacy in the treatment of schizophrenia symptoms.^30^ A meta-analysis of 40 randomized controlled trials (RCTs) showed that as an adjunctive treatment to antipsychotics, glycine site NMDAR coagonists, including D-serine, D-cycloserine, and glycine, improved schizophrenia symptoms with significant effect sizes.^30^ One RCT found that the improvement in clinical symptoms of schizophrenia observed with D-serine treatment was accompanied by a significant improvement in MMN.^31^ This is in alignment with evidence suggesting that serum D-serine levels are considerably reduced in patients with schizophrenia compared with healthy individuals,^32^ and that improved cognitive functioning was associated with increased serum D-serine levels in patients with schizophrenia.^33,34^

As one of the more extensively studied NMDAR agonists, D-serine has been scrutinized from a safety perspective because nephrotoxicity was previously observed in rats treated with high-dose D-serine.^35^ High doses of exogenous D-serine are required for efficacy, in part because it has a relatively short half-life in humans,^36^ likely due to rapid metabolism by kidney D-amino acid oxidase (DAAO).^36,37^ Nephrotoxicity has not been observed in humans, except for a single participant who had mildly abnormal renal values when treated with high-dose D-serine (120 mg/kg).^35,36^ All subsequent trials of D-serine (with the exception of the 2 RCTs mentioned hereafter) utilized low doses (≤30 mg/kg) and observed modest effect sizes for treating schizophrenia symptoms^35^; however, higher doses of D-serine (≥60 mg/kg) appear to be more effective at improving symptoms than low doses in humans and mice.^31,35,36^ Despite this initial promise, the clinical development of high-dose D-serine has not progressed beyond phase 2 studies.

In addition to the D-serine studies, a similar number of small RCTs have investigated glycine as a potential treatment for people with schizophrenia. Glycine has poor blood-brain penetration; thus, high doses are required to achieve efficacy.^38^ A meta-analysis of 40 RCTs investigating NMDAR modulators, 11 of which studied glycine, showed an overall reduction in negative symptoms with glycine treatment.^30^ However, glycine treatment did not show any significant efficacy for negative symptoms in the CONSIST trial (the largest to date, with *N* = 165 randomized participants, 54 of whom were assigned to glycine),^39^ in the longest glycine trial reported (28 weeks),^40^ and in one of the most recent glycine trials in participants with schizophrenia and alcohol dependence.^41^ Furthermore, similarly to D-serine, glycine has not yet shown efficacy in any large phase 3 RCTs.

Given these mixed outcomes with D-serine and glycine, as well as the high doses required for efficacy, an alternative approach to increase endogenous D-serine or glycine levels in the human brain was sought, resulting in the development of indirect glycine site modulators. As discussed in Section 3, DAAO inhibitors, which function by indirectly increasing D-serine in the human brain, have emerged as novel potential alternative treatments for patients with schizophrenia (Figure 2B). In a similar indirect manner, glycine transporter 1 (GlyT1) inhibitors function by increasing glycine levels. Both DAAO inhibitors and GlyT1 inhibitors are under clinical development; to date, none are approved for the treatment of schizophrenia.

## DAAO INHIBITORS

In mammals, DAAO is the primary enzyme that degrades and eliminates the amino acid D-serine in the central nervous system (CNS).^42^ In rats, DAAO expression in the CNS is confined to the brain stem and cerebellum,^43^ while in humans it is located in the cerebellum, prefrontal cortex, hippocampus, and substantia nigra.^44^ Given the relationship between DAAO and D-serine, it follows that inhibiting DAAO will cause D-serine levels in the CNS to increase. Studies from over a decade ago demonstrated that mice lacking DAAO had increased D-serine levels, increased NMDAR activity, enhanced spatial learning, and a general lack of negative physiological effects.^45-47^ The role that these elements play in schizophrenia suggests that DAAO inhibition may be a plausible treatment pathway for this illness.

In addition to the target validation of DAAO provided by the studies in genetically modified mice, studies of sodium benzoate have provided further evidence that DAAO inhibitors may effectively treat schizophrenia. Sodium benzoate is a low-potency DAAO inhibitor that requires relatively high doses to achieve sufficient DAAO enzyme occupancy.^48,49^ In human trials, the first small RCT (*n* = 52) to study the potential efficacy of sodium benzoate as an adjunctive treatment in people with schizophrenia reported improved negative symptoms and neurocognition,^50^ but 3 subsequent sodium benzoate studies in specific patient populations showed mixed results in improving schizophrenia symptomatology. In a small RCT of patients with schizophrenia (*n* = 60) stabilized on clozapine, but with residual symptoms, sodium benzoate showed efficacy for positive, negative, and total symptoms, but no effect on cognition.^51^ As an add-on treatment to sarcosine in patients with chronic schizophrenia (*n* = 42), sodium benzoate improved cognitive functioning but not positive, negative, or total symptoms.^52^ Finally, another small RCT (*n* = 100) did not show efficacy in participants with early psychosis, the majority of whom had schizophrenia; of note, cognitive endpoints were not included in this study.^49^ One complicating factor in interpreting these studies is that, while the relationship between DAAO inhibition and D-serine is well established, the increase in cerebellar and plasma D-serine levels resulting from sodium benzoate treatment in mice^48^ has not been replicated in humans.^49,52^ However, despite this lack of a measurable change in D-serine levels due to sodium benzoate, there is evidence that sodium benzoate increases plasma catalase activity^52,53^ and alters fMRI-measurable brain circuits,^54^ indicating sodium benzoate pharmacological activity in humans. Although these studies have not led to more widespread use of sodium benzoate as an adjunctive therapy for patients with schizophrenia, they supported further development of DAAO inhibition as a potential pharmacological target for the treatment of schizophrenia.

Promising data from mice genetically altered to lack DAAO, combined with the initial hints of efficacy with the DAAO inhibitor sodium benzoate, stimulated considerable interest in the development of more potent, selective, and drug-like DAAO inhibitors, with several pharmaceutical companies designing novel human DAAO inhibitors.^55-58^ Many of these DAAO inhibitors contain a carboxylic acid group that can form a strong interaction with the DAAO active site arginine^59^; however, the presence of a negatively charged carboxylic acid group or other larger functional groups can negatively affect the ability of these compounds to penetrate the blood-brain barrier.^59^ Furthermore, the relatively small active site of the DAAO enzyme limited the diversity of compounds that medicinal chemists could explore as DAAO inhibitors.^59^ It has been a significant challenge to design and develop compounds that could effectively engage the DAAO active site while maintaining the necessary oral bioavailability, brain penetration, and pharmacokinetic (PK) stability required to progress to efficacy studies in patients.^59^

## LUVADAXISTAT

Luvadaxistat (TAK-831, NBI-1065844) is an investigational, highly selective, and potent DAAO inhibitor^60^ that has been shown to increase D-serine levels in the cerebellum, plasma, and cerebrospinal fluid (CSF) of rodents and also in the plasma and CSF of participants in phase 1 studies.^61,62^ The following sections summarize the pharmacology of luvadaxistat in rodents, pharmacology and biomarker effects in healthy volunteers, and efficacy in a randomized, placebo-controlled phase 2 study in schizophrenia. Collectively, these data highlight key elements of the mechanism of action of luvadaxistat that provided the rationale for potential as a treatment for CIAS.

### Pharmacology of Luvadaxistat in Rodents

While the complexity of the cognitive and negative symptoms of schizophrenia cannot be fully embodied by rodent models, luvadaxistat has been tested in various rodent paradigms that have explored cognitive, behavioral, and neurocircuitry function thought to be related to schizophrenia.^60^ In cognitive tasks, luvadaxistat treatment enhanced learning and memory in both the novel object recognition (NOR) task and in the PCP-impairment version of the attentional set shifting task (ASST).^60^ While both acute and chronic dosing regimens were effective, luvadaxistat was more potent (ie, effective at lower doses) in both the NOR task and ASST when administered chronically, suggesting a sensitization effect of DAAO inhibition. In the NOR task, chronic dosing was also associated with an inverted U-shaped dose-response curve. Specifically, while luvadaxistat was efficacious in the NOR task at low/moderate doses (0.003 mg/kg to 0.1 mg/kg), there was no efficacy at the highest doses tested (0.3 mg/kg and 1 mg/kg).^60^ Interestingly, luvadaxistat enhanced cognition in the ASST when administered with or without haloperidol.

Social withdrawal and social avoidance are aspects of negative symptoms in people with schizophrenia.^3^ To address domains of rodent behavior that are potentially relevant to the negative symptoms of schizophrenia, luvadaxistat was tested in 2 rodent models of social behavior. The BALB/c (Bagg Albino) mouse strain, due to inherent susceptibility to stress, is often used to assess rodent social behavior impairment.^63^ In this assay, luvadaxistat, dosed either acutely or chronically (14 days), increased interaction of the dosed mouse with an “intruder” mouse, suggesting a normalization of natural rodent behavior.^60^ Mirroring the pharmacology described above for the NOR task, luvadaxistat showed a sensitization effect, in which it was more potent when dosed chronically than when dosed acutely. In a second model of rodent social behavior, C57BL/6 mice were treated with polyinosinic:polycytidylic acid to produce a social impairment (ie, less engagement of an intruder mouse).^60^ This impairment was normalized by acute luvadaxistat treatment.

It has been postulated that cerebellar anomalies may be associated with schizophrenia^64^ and, as previously mentioned, the cerebellum is thought to express high levels of DAAO mRNA and protein^43,44^; consequently, luvadaxistat was tested in a functional measure of cerebellar activity. Specifically, cerebellar-mediated learning processes can be assessed with an eyeblink conditioning test in animal models.^65^ Furthermore, because similar measures can be used in people, eyeblink conditioning is a candidate translational biomarker.^65^ In the mouse eyeblink conditioning test, luvadaxistat was efficacious when dosed chronically for 10 days.^60^

Long-term potentiation (LTP), as measured in rodent hippocampal slices, has 2 features rendering it a valuable experimental model in which to explore DAAO inhibitor function: (1) LTP is thought to be a cellular/circuitry correlate of learning and memory and (2) the NMDAR is an important component of LTP induction in the hippocampus.^66,67^ Luvadaxistat was studied in LTP models in which mice were dosed with a test compound in vivo; 5 hours later, mice were sacrificed, and LTP was tested in slices ex vivo. In these studies, acute doses of luvadaxistat had no measurable effects on LTP; however, after 14 days of subchronic daily dosing of luvadaxistat, NMDAR-dependent LTP was enhanced. Consistent with the effects in NOR described above, an inverted U-shaped dose-response curve was observed: higher doses (0.1 mg/kg and 10 mg/kg) decreased LTP, while low doses of luvadaxistat (0.001 mg/kg and 0.01 mg/kg) increased LTP, with the 0.01 mg/kg dose demonstrating the largest increase.^60^

A mechanistic multilayer quantitative model, established in mice, has been used to understand better the relationship between the biodistribution of luvadaxistat and subsequent functional effects.^68^ This model utilized data relating to PK, DAAO occupancy, and pharmacodynamics (PD; measuring the increase in cerebellar D-serine levels) of luvadaxistat. The model highlighted the slow binding kinetics of luvadaxistat, showing that luvadaxistat treatment would yield sustained DAAO inhibition beyond what would be predicted by the PK profile. Furthermore, these data showed that luvadaxistat was more potent in vivo than was predicted based on in vitro studies; this phenomenon is often driven by a slow off rate, which produces sustained effects.

In summary, luvadaxistat has demonstrated efficacy in various rodent models of cognitive function and social behavior. Although it is well established that these assays have limited predictive validity to human diseases, the results provide some evidence that luvadaxistat engages brain circuits relevant to the pathophysiology of schizophrenia. Four rodent pharmacological assays tested multiple doses of luvadaxistat, administered both acutely and subchronically (Table 1).^60^ In all 4 of these models, there was a sensitization effect, in which subchronic dosing was more potent than acute dosing. This observation is consistent with luvadaxistat engaging plasticity mechanisms, which over multiple days of dosing led to enhanced potency compared with a single dose. Furthermore, the results from Yoneyama et al.^68^ demonstrate that the effects of luvadaxistat are sustained compared to what was predicted by its PK profile. Finally, in 2 of 4 rodent assays, luvadaxistat exhibited an inverted U-shaped dose-response curve, in which efficacy is lost at the highest doses tested. These features of sensitization, sustained effect, and inverted U-shaped response curve demonstrate that luvadaxistat has a complex pharmacology; this complexity informs subsequent PK/PD results when tested in humans.

**Table 1.** Luvadaxistat activity in animal models tested using acute and subchronic dosing.

Rodent assay	Sensitization?	Inverted U?
NOR	Yes	Yes
ASST	Yes	No
LTP	Yes	Yes
Social interaction (BALB/c)	Yes	No

Acute dosing was a single dose, while subchronic dosing of luvadaxistat involved multiple doses over days or weeks (Fradley et al. ^60^). In these assays, “Yes” under “Sensitization?” means that the subchronic dosing regimen was more potent than acute dosing. “Yes” under “Inverted U-shaped dose response?” means that efficacy was observed at lower doses but did not occur at higher doses.

Abbreviations: ASST, attentional set shifting task; LTP, long-term potentiation; NOR, novel object recognition.

### Pharmacology of Luvadaxistat in Humans

Two phase 1 clinical trials (NCT02566759 and NCT03224325) assessed the safety, tolerability, PK, and PD of single and multiple oral doses of luvadaxistat.^69^ All doses were well tolerated, with nausea and headaches reported more frequently by participants receiving luvadaxistat than by those receiving placebo. Luvadaxistat was absorbed rapidly following oral administration, with plasma concentrations reaching maximal levels within a median of 0.25–2 hours after dosing; systemic exposures also showed dose-dependent increases after single and multiple dosing. The mean terminal half-life for luvadaxistat ranged from 6 hours to 23 hours, aligning with the observation that there is minimal accumulation of luvadaxistat after daily dosing. Notably, a single dose of luvadaxistat resulted in elevated D-serine levels in the plasma and CSF of healthy volunteers, which persisted for 24 hours. This implies that once-daily dosing of luvadaxistat would yield a sustained elevation of D-serine, reflecting the sustained effect observed in preclinical studies.^68^ Furthermore, data from a positron emission tomography study confirmed that, at tolerable doses, luvadaxistat binds to DAAO at nearly full occupancy (data not published; NCT02716987), while PK/PD modeling has predicted that low doses of luvadaxistat have a DAAO enzyme occupancy of 50%-90%.^70^

Neither low nor high doses of luvadaxistat affected cerebellar-dependent learning as measured by eyeblink conditioning in a phase 2a biomarker-focused, placebo-controlled study (NCT03359785).^71^ Although these data should be interpreted carefully owing to the small sample size, this result does call into question the translatability of this paradigm because, as described above, luvadaxistat displayed efficacy in the mouse version of this paradigm.^60^ In the phase 2a biomarker study, a nominally significant improvement in MMN and a statistical trend toward improvement for the ASSR were observed.^71^ This result is consistent with enhanced NMDAR activity and suggests that luvadaxistat treatment has the potential to improve the auditory processing circuits that are impaired in schizophrenia.^72-74^ Furthermore, this finding is reminiscent of the improvements in MMN observed with D-serine treatment, as described in Section 2. Similar to the results observed in rodent studies (Table 1), the improvements in MMN and ASSR observed in O’Donnell, Dong^71^ followed an inverted U-shaped dose-response, with effects observed at the 50 mg, but not 500 mg, dose of luvadaxistat.

### Efficacy and Safety of Luvadaxistat in Humans (Phase 2 INTERACT Study; NCT03382639)

**Figure 3.**
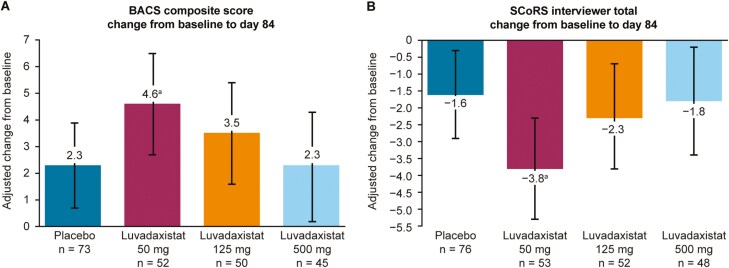
LS mean change in cognitive performance and function from baseline to day 84 measured by (A) the BACS composite score and (B) the SCoRS interviewer total score. Data were analyzed using the MMRM model, based on a missing at random assumption, using observed case data only. One-sided *P*-value versus placebo: <.05. Error bars show 95% confidence intervals. ^a^ Change from baseline to day 84 in BACS composite score and SCoRS interviewer total score both had an effect size of 0.4 for 50 mg versus placebo. Abbreviations: BACS, Brief Assessment of Cognition in Schizophrenia; LS, least squares; MMRM, mixed model for repeated measures; SCoRS, Schizophrenia Cognition Rating Scale. Figure modified from Murthy et al. *Schizophr Res* 2024; **270**:249–57^75^ with permission under the terms of the CC BY 4.0 license (https://creativecommons.org/licenses/by/4.0/) to show error bars as 95% confidence intervals instead of standard error.

INTERACT was a phase 2, randomized, placebo-controlled study that investigated the efficacy and safety of 3 doses of luvadaxistat (50, 125, and 500 mg) in participants with schizophrenia who had persistent negative symptoms.^75^ Although INTERACT did not meet the primary endpoint of a change from baseline in the Positive and Negative Syndrome Scale Negative Symptom Factor Score (PANSS NSFS), it did meet the prespecified secondary endpoints for cognition, with nominally significant improvements in cognitive performance (the Brief Assessment of Cognition in Schizophrenia [BACS] composite score) and cognitive function (the Schizophrenia Cognition Rating Scale [SCoRS] interviewer, informant, and global rating scores) for the luvadaxistat 50 mg group compared with placebo (Figure 3).^75^ Notably, high study medication adherence (measured using a digital health technology tool) was observed in both placebo and luvadaxistat treatment groups. The improvements in BACS and SCoRS scores were associated with study medication adherence in those receiving luvadaxistat.^75-78^ Aligning with rodent pharmacology (Table 1) and the suggestions from the biomarker study,^71^ luvadaxistat treatment resulted in an inverted U-shaped dose-response curve, with clinically meaningful improvements in cognition and cognitive functional outcomes observed at the lower 50 mg dose, but not at the higher 125 mg and 500 mg doses.^75^ Finally, luvadaxistat treatment was well tolerated and no new safety signals were identified at any dose.^75^ Given the potential effect on cognition that was observed in the INTERACT study, luvadaxistat was assessed in the phase 2 ERUDITE study as an investigational drug for treating patients with CIAS (CT.gov; NCT05182476). However, the ERUDITE study failed to replicate the cognitive endpoints data seen in the INTERACT study, partly due to the large variability seen in the cognitive measures across the population studied and a potential imbalance in the baseline characteristics of participants across the treatment arms.^79^

It is worth noting that these findings are similar to those seen in studies of GlyT1 inhibitors, which are also postulated to increase NMDAR activity via the accumulation of glycine. Although the GlyT1 inhibitor bitopertin achieved the primary outcome of a change from baseline in the PANSS NSFS score in a phase 2 trial that recruited participants with schizophrenia and predominant negative symptoms,^80^ it did not achieve this improvement in negative symptoms in phase 3 trials.^81^ More recently, treatment with the GlyT1 inhibitor iclepertin was shown to improve cognition in participants with schizophrenia, as assessed by the MATRICS Consensus Cognitive Battery (MCCB) overall composite T-score in a phase 2 study; however, iclepertin treatment did not show efficacy in addressing negative symptoms.^82^ Because computerized cognitive treatment (CCT) has shown some promise as a non-pharmacological treatment for CIAS,^83^ there was interest in determining if the combination of iclepertin with CCT would yield additional benefits for patients. A phase 2 study of 200 participants with schizophrenia (NCT03859973) compared treatment with iclepertin 10 mg in combination with CCT to placebo combined with CCT. The results posted on govclinicaltrialsregister.eu (EudraCT number: 2018-002740-82) showed no statistical improvement in MCCB and SCoRS scores with the combination of iclepertin-CCT compared to placebo-CCT at the 12-week endpoint. Given the improvement in cognition that was observed when iclepertin was used alone against placebo, iclepertin is being investigated in phase 3 trials as a potential adjunctive therapy for patients with CIAS.^84^

## DAAO VS GLYT1 INHIBITION CONSIDERATIONS

Throughout this review, we have considered DAAO inhibitors and GlyT1 inhibitors as having roughly the same functional effects: DAAO inhibitors elevate brain D-serine and GlyT1 inhibitors elevate brain glycine, with both activities generally promoting NMDAR activity. However, there are notable differences between DAAO/D-serine and GlyT1/glycine. Depending on whether activity, protein, or mRNA is assessed in rodents or humans, DAAO has been shown to be enriched in the cerebellum and other hindbrain regions, with a substantial amount of DAAO protein also found in the cortex and hippocampus.^59^ Focusing on GlyT1, human positron emission tomography (PET) studies reveal the highest expression in the pons and thalamus, moderate expression in the striatum and hippocampus, and lower expression throughout the cortex.^85^ Although D-serine and glycine are coagonists at the NR2 subunit, they have differential affinities for the variants of this subunit,^86-89^ which may in part explain the differential effects of glycine and D-serine on glutamatergic transmission.^90^ Furthermore, studies also suggest that D-serine acts predominantly at synaptic NMDARs, whereas glycine acts at extrasynaptic NMDARs.^91,92^ In the face of the ERUDITE study, in which luvadaxistat failed as a potential treatment for CIAS,^79^ should the phase 3 trial of the GlyT1 inhibitor iclepertin as a treatment for CIAS be successful, these cellular differences would be of great interest as a potential reason underlying the differences seen in efficacy with DAAO inhibitors versus GlyT1 inhibitors in treating CIAS.

To date, DAAO inhibitors and GlyT1 inhibitors that have shown positive efficacy signals in early phase 2 trials have failed to replicate these signals in larger phase 2 and phase 3 trials. Given that glycine, D-serine, GlyT1 inhibitors, and DAAO inhibitors have all shown functional effects in rodent and human studies, as described throughout this review, these negative studies may simply result from the specific challenges that come with measuring cognition in patients with schizophrenia, in addition to typical clinical trial conduct challenges in patients with mental illness, such as ensuring medication adherence. As another potential explanation for the lack of reproducible efficacy for this class of drug candidates, although occupancy of the NMDAR glycine site varies across synapses throughout the brain,^93^ NMDAR glycine site occupancy is very high or even saturated at baseline in certain synapses.^94^ High baseline occupancy, coupled with evidence that engagement of the NMDAR glycine site approaching 100% can lead to clathrin-mediated NMDAR internalization,^95,96^ may suggest that there is only a narrow window to observe NMDAR functional effects, as has been demonstrated in clinical trials for luvadaxistat, bitopertin, and iclepertin.

Guided by the NMDAR hypofunction model of schizophrenia, most clinical trials have focused on cognitive impairment, or other symptom domains, in schizophrenia. However, the NMDAR is implicated in multiple diseases, and the low-potency DAAO inhibitor sodium benzoate has demonstrated efficacy in improving cognition in disease states other than CIAS. Studies have shown that sodium benzoate treatment improved cognitive impairment in Alzheimer’s disease and in patients with behavioral and psychological symptoms of dementia,^97,98^ with a post hoc analysis of the latter study demonstrating significant efficacy only in women.^97^ Sodium benzoate also improved cognitive function and perceived stress in late-life depression.^99^

Finally, it is well understood that schizophrenia and other mental illnesses are highly heterogenous. Many of the genetic variants linked to schizophrenia risk implicate NMDARs and other glutamate signaling proteins, and recent efforts have attempted to link these genetic variants to functional measures of the glutamate system in humans.^100^ Collectively, these data suggest that DAAO inhibitors, and other NMDAR modulators, could impact neuronal function differentially in patients with different genetic backgrounds. Future efforts could focus on determining genetic or functional biomarkers, which could subsequently identify patients who would derive the most benefit from treatment with NMDAR modulators.

## SUMMARY

In line with the NMDAR hypofunction model of schizophrenia, this review has summarized current evidence supporting NMDAR agonism at the glycine site as a potentially effective treatment for patients with schizophrenia. Clinical experience with indirect modulators of the NMDAR glycine site, such as luvadaxistat (DAAO inhibitor) and iclepertin (GlyT1 inhibitor), demonstrates that these compounds enhance cognition in measurable ways in phase 2 studies. However, the therapeutic effects, if any, on negative or cognitive symptoms have been difficult to establish convincingly and reproducibly in modern study designs in patients with schizophrenia. There are numerous reasons that potentially underlie the inability to convert the mechanistic promise of DAAO inhibitors and GlyT1 inhibitors into FDA-approved medications. Resolving this impasse may be achievable in future studies by carefully selecting the patient population (perhaps guided by biomarkers), precisely selecting the specific disease indication, refining clinical trial conduct, or other strategies not outlined in this review, such as well-considered drug combination protocols. Any initial, convincing drug approval success could then serve as a springboard to guide further investment, innovation, and refinement of the strategies needed to observe therapeutic benefit from this class of compounds.

## Data Availability

All data are available on request.

## References

[1] Global Burden of Disease. Global, regional, and national incidence, prevalence, and years lived with disability for 328 diseases and injuries for 195 countries, 1990-2016: a systematic analysis for the Global Burden of Disease Study 2016. Lancet. 2016;390:1211-1259. 10.1016/S0140-6736(17)32154-2 PMC5605509 28919117

[2] McCutcheon RA, Keefe RSE, McGuire PK. Cognitive impairment in schizophrenia: aetiology, pathophysiology, and treatment. Mol Psychiatry. 2023;28:1902-1918. https://doi.org/10.1038/s41380-023-01949-9 36690793 PMC10575791

[3] Mosolov SN, Yaltonskaya PA. Primary and secondary negative symptoms in schizophrenia. Front Psychiatry. 2021;12:766692. https://doi.org/10.3389/fpsyt.2021.766692 35046851 PMC8761803

[4] Sommer IE, Bearden CE, van Dellen E, et al Early interventions in risk groups for schizophrenia: what are we waiting for? NPJ Schizophr. 2016;2:16003. https://doi.org/10.1038/npjschz.2016.3 27336054 PMC4849435

[5] Murray AJ, Rogers JC, Katshu M, Liddle PF, Upthegrove R. Oxidative stress and the pathophysiology and symptom profile of schizophrenia spectrum disorders. Front Psychiatry. 2021;12:703452. 34366935 10.3389/fpsyt.2021.703452 PMC8339376

[6] Nakazawa K, Zsiros V, Jiang Z, et al GABAergic interneuron origin of schizophrenia pathophysiology. Neuropharmacology. 2012;62:1574-1583. https://doi.org/10.1016/j.neuropharm.2011.01.022 21277876 PMC3090452

[7] Diagnostic and Statistical Manual of Mental Disorders 5th ed. The Diagnostic and Statistical Manual of Mental Disorders. 5th ed.; DSM–5. American Psychiatric Association; 2013.

[8] Zaragoza Domingo S, Bobes J, Garcia-Portilla MP, Morralla C; EPICOG-SCH Study Group. Cognitive Performance associated to functional outcomes in stable outpatients with schizophrenia. Schizophr Res Cogn. 2015;2:146-158. https://doi.org/10.1016/j.scog.2015.03.002 29379764 PMC5779297

[9] Bobes J, Arango C, Garcia-Garcia M, Rejas J; Clamors Study Collaborative Group. Prevalence of negative symptoms in outpatients with schizophrenia spectrum disorders treated with antipsychotics in routine clinical practice: findings from the CLAMORS study. J Clin Psychiatry. 2010;71:280-286. 19895779 10.4088/JCP.08m04250yel

[10] Shamsi S, Lau A, Lencz T, et al Cognitive and symptomatic predictors of functional disability in schizophrenia. Schizophr Res. 2011;126:257-264. https://doi.org/10.1016/j.schres.2010.08.007 20828991 PMC3050077

[11] Mucci A, Galderisi S, Gibertoni D, et al Italian Network for Research on Psychoses Factors associated with real-life functioning in persons with schizophrenia in a 4-year follow-up study of the Italian Network for research on psychoses. JAMA Psychiatry 2021;78:550-559. https://doi.org/10.1001/jamapsychiatry.2020.4614 33566071 PMC7876615

[12] Galderisi S, Rossi A, Rocca P, et al Italian Network For Research on Psychoses The influence of illness-related variables, personal resources and context-related factors on real-life functioning of people with schizophrenia. World Psychiatry 2014;13:275-287. https://doi.org/10.1002/wps.20167 25273301 PMC4219069

[13] Kaar SJ, Natesan S, McCutcheon R, Howes OD. Antipsychotics: mechanisms underlying clinical response and side-effects and novel treatment approaches based on pathophysiology. Neuropharmacology. 2020;172:107704. https://doi.org/10.1016/j.neuropharm.2019.107704 31299229

[14] Olney JW, Newcomer JW, Farber NB. NMDA receptor hypofunction model of schizophrenia. J Psychiatr Res. 1999;33:523-533. https://doi.org/10.1016/s0022-3956(99)00029-1 10628529

[15] Jentsch JD, Roth RH. The neuropsychopharmacology of phencyclidine: from NMDA receptor hypofunction to the dopamine hypothesis of schizophrenia. Neuropsychopharmacol. 1999;20:201-225. https://doi.org/10.1016/S0893-133X(98)00060-8 10063482

[16] Kayser MS, Dalmau J. Anti-NMDA receptor encephalitis, autoimmunity, and psychosis. Schizophr Res. 2016;176:36-40. https://doi.org/10.1016/j.schres.2014.10.007 25458857 PMC4409922

[17] Dalmau J, Tüzün E, Wu H-Y, et al Paraneoplastic anti-N-methyl-D-aspartate receptor encephalitis associated with ovarian teratoma. Ann Neurol. 2007;61:25-36. https://doi.org/10.1002/ana.21050 17262855 PMC2430743

[18] Dalmau J, Gleichman AJ, Hughes EG, et al Anti-NMDA-receptor encephalitis: case series and analysis of the effects of antibodies. Lancet Neurol. 2008;7:1091-1098. https://doi.org/10.1016/S1474-4422(08)70224-2 18851928 PMC2607118

[19] Schizophrenia Working Group of the Psychiatric Genomics Consortium. Biological insights from 108 schizophrenia-associated genetic loci. Nature. 2014;511:421-427. 25056061 10.1038/nature13595 PMC4112379

[20] Ohi K, Hashimoto R, Ikeda M, et al Glutamate networks implicate cognitive impairments in schizophrenia: genome-wide association studies of 52 cognitive phenotypes. Schizophr Bull. 2015;41:909-918. https://doi.org/10.1093/schbul/sbu171 25537281 PMC4466179

[21] Light GA, Braff DL. Mismatch negativity deficits are associated with poor functioning in schizophrenia patients. Arch Gen Psychiatry. 2005;62:127-136. https://doi.org/10.1001/archpsyc.62.2.127 15699289

[22] Thune H, Recasens M, Uhlhaas PJ. The 40-Hz auditory steady-state response in patients with schizophrenia: a meta-analysis. JAMA Psychiatry. 2016;73:1145-1153. https://doi.org/10.1001/jamapsychiatry.2016.2619 27732692

[23] Catts VS, Lai YL, Weickert CS, Weickert TW, Catts SV. A quantitative review of the postmortem evidence for decreased cortical N-methyl-D-aspartate receptor expression levels in schizophrenia: how can we link molecular abnormalities to mismatch negativity deficits? Biol Psychol. 2016;116:57-67. https://doi.org/10.1016/j.biopsycho.2015.10.013 26549579

[24] Glausier JR, Lewis DA. Dendritic spine pathology in schizophrenia. Neuroscience. 2013;251:90-107. https://doi.org/10.1016/j.neuroscience.2012.04.044 22546337 PMC3413758

[25] Traynelis SF, Wollmuth LP, McBain CJ, et al Glutamate receptor ion channels: structure, regulation, and function. Pharmacol Rev. 2010;62:405-496. https://doi.org/10.1124/pr.109.002451 20716669 PMC2964903

[26] Campanini B, Spyrakis F, Peracchi A, Mozzarelli A. Serine racemase: a key player in neuron activity and in neuropathologies. Front Biosci (Landmark Ed). 2013;18:1112-1128. https://doi.org/10.2741/4167 23747871

[27] Martineau M, Parpura V, Mothet JP. Cell-type specific mechanisms of D-serine uptake and release in the brain. Front Synaptic Neurosci. 2014;6:12. https://doi.org/10.3389/fnsyn.2014.00012 24910611 PMC4039169

[28] Van Horn MR, Sild M, Ruthazer ES. D-serine as a gliotransmitter and its roles in brain development and disease. Front Cell Neurosci. 2013;7:39. https://doi.org/10.3389/fncel.2013.00039 23630460 PMC3632749

[29] Rosenberg D, Artoul S, Segal AC, et al Neuronal D-serine and glycine release via the Asc-1 transporter regulates NMDA receptor-dependent synaptic activity. J Neurosci. 2013;33:3533-3544. https://doi.org/10.1523/JNEUROSCI.3836-12.2013 23426681 PMC6619521

[30] Goh KK, Wu TH, Chen CH, Lu ML. Efficacy of N-methyl-D-aspartate receptor modulator augmentation in schizophrenia: a meta-analysis of randomised, placebo-controlled trials. J Psychopharmacol. 2021;35:236-252. https://doi.org/10.1177/0269881120965937 33406959

[31] Kantrowitz JT, Epstein ML, Lee M, et al Improvement in mismatch negativity generation during D-serine treatment in schizophrenia: correlation with symptoms. Schizophr Res. 2018;191:70-79. https://doi.org/10.1016/j.schres.2017.02.027 28318835

[32] Cho SE, Na KS, Cho SJ, Kang SG. Low D-serine levels in schizophrenia: a systematic review and meta-analysis. Neurosci Lett. 2016;634:42-51. https://doi.org/10.1016/j.neulet.2016.10.006 27717827

[33] Panizzutti R, Fisher M, Garrett C, et al Association between increased serum d-serine and cognitive gains induced by intensive cognitive training in schizophrenia. Schizophr Res. 2019;207:63-69. https://doi.org/10.1016/j.schres.2018.04.011 29699895 PMC9770102

[34] Hons J, Zirko R, Vasatova M, et al Impairment of executive functions associated with lower D-serine serum levels in patients with schizophrenia. Front Psychiatry. 2021;12:514579. https://doi.org/10.3389/fpsyt.2021.514579 33854443 PMC8039447

[35] Meftah A, Hasegawa H, Kantrowitz JT. D-Serine: a cross species review of safety. Front Psychiatry. 2021;12:726365. https://doi.org/10.3389/fpsyt.2021.726365 34447324 PMC8384137

[36] Kantrowitz JT, Malhotra AK, Cornblatt B, et al High dose D-serine in the treatment of schizophrenia. Schizophr Res. 2010;121:125-130. https://doi.org/10.1016/j.schres.2010.05.012 20541910 PMC3111070

[37] Rais R, Thomas AG, Wozniak K, et al Pharmacokinetics of oral D-serine in D-amino acid oxidase knockout mice. Drug Metab Dispos. 2012;40:2067-2073. https://doi.org/10.1124/dmd.112.046482 22837388 PMC3477206

[38] Javitt DC, Silipo G, Cienfuegos A, et al Adjunctive high-dose glycine in the treatment of schizophrenia. Int J Neuropsychopharmacol. 2001;4:385-391. https://doi.org/10.1017/S1461145701002590 11806864

[39] Buchanan RW, Javitt DC, Marder SR, et al The Cognitive and Negative Symptoms in Schizophrenia Trial (CONSIST): the efficacy of glutamatergic agents for negative symptoms and cognitive impairments. Am J Psychiatry. 2007;164:1593-1602. https://doi.org/10.1176/appi.ajp.2007.06081358 17898352

[40] Diaz P, Bhaskara S, Dursun SM, Deakin B. Double-blind, placebo-controlled, crossover trial of clozapine plus glycine in refractory schizophrenia negative results. J Clin Psychopharmacol. 2005;25:277-278. https://doi.org/10.1097/01.jcp.0000165740.22377.6d 15876911

[41] Serrita J, Ralevski E, Yoon G, Petrakis I. A pilot randomized, placebo-controlled trial of glycine for treatment of schizophrenia and alcohol dependence. J Dual Diagn. 2019;15:46-55. https://doi.org/10.1080/15504263.2018.1549764 30633660

[42] Molla G. Competitive inhibitors unveil structure/function relationships in human D-amino acid oxidase. Front Mol Biosci. 2017;4:80. https://doi.org/10.3389/fmolb.2017.00080 29250527 PMC5715370

[43] Horiike K, Tojo H, Arai R, Nozaki M, Maeda T. D-amino-acid oxidase is confined to the lower brain stem and cerebellum in rat brain: regional differentiation of astrocytes. Brain Res. 1994;652:297-303. https://doi.org/10.1016/0006-8993(94)90240-2 7953743

[44] Verrall L, Walker M, Rawlings N, et al d-Amino acid oxidase and serine racemase in human brain: normal distribution and altered expression in schizophrenia. Eur J Neurosci. 2007;26:1657-1669. https://doi.org/10.1111/j.1460-9568.2007.05769.x 17880399 PMC2121142

[45] Almond SL, Fradley RL, Armstrong EJ, et al Behavioral and biochemical characterization of a mutant mouse strain lacking D-amino acid oxidase activity and its implications for schizophrenia. Mol Cell Neurosci. 2006;32:324-334. https://doi.org/10.1016/j.mcn.2006.05.003 16843004

[46] Maekawa M, Watanabe M, Yamaguchi S, Konno R, Hori Y. Spatial learning and long-term potentiation of mutant mice lacking D-amino-acid oxidase. Neurosci Res. 2005;53:34-38. https://doi.org/10.1016/j.neures.2005.05.008 15996778

[47] Yamanaka M, Miyoshi Y, Ohide H, Hamase K, Konno R. D-Amino acids in the brain and mutant rodents lacking D-amino-acid oxidase activity. Amino Acids. 2012;43:1811-1821. https://doi.org/10.1007/s00726-012-1384-x 22892863

[48] Howley E, Bestwick M, Fradley R, et al Assessment of the target engagement and D-serine biomarker profiles of the D-amino acid oxidase inhibitors sodium benzoate and PGM030756. Neurochem Res. 2017;42:3279-3288. https://doi.org/10.1007/s11064-017-2367-9 28780732

[49] Scott JG, Baker A, Lim CCW, et al Effect of sodium benzoate vs placebo among individuals with early psychosis: a randomized clinical trial. JAMA Netw Open. 2020;3:e2024335. https://doi.org/10.1001/jamanetworkopen.2020.24335 33170261 PMC7656289

[50] Lane HY, Lin C-H, Green MF, et al Add-on treatment of benzoate for schizophrenia: a randomized, double-blind, placebo-controlled trial of D-amino acid oxidase inhibitor. JAMA Psychiatry. 2013;70:1267-1275. https://doi.org/10.1001/jamapsychiatry.2013.2159 24089054

[51] Lin CH, Lin C-H, Chang Y-C, et al Sodium benzoate, a D-amino acid oxidase inhibitor, added to clozapine for the treatment of schizophrenia: a randomized, double-blind, placebo-controlled trial. Biol Psychiatry. 2018;84:422-432. https://doi.org/10.1016/j.biopsych.2017.12.006 29397899

[52] Lin CY, Liang S-Y, Chang Y-C, et al Adjunctive sarcosine plus benzoate improved cognitive function in chronic schizophrenia patients with constant clinical symptoms: a randomised, double-blind, placebo-controlled trial. World J Biol Psychiatry. 2017;18:357-368. https://doi.org/10.3109/15622975.2015.1117654 26691576

[53] Lane HY, Wang SH, Lin CH. Sex- and dose-dependent catalase increase and its clinical impact in a benzoate dose-finding, randomized, double-blind, placebo-controlled trial for Alzheimer’s disease. Pharmacol Biochem Behav. 2024;245:173885. https://doi.org/10.1016/j.pbb.2024.173885 39384087

[54] Lane HY, Tu CH, Lin WC, Lin CH. Brain activity of benzoate, a D-amino acid oxidase inhibitor, in patients with mild cognitive impairment in a randomized, double-blind, placebo controlled clinical trial. Int J Neuropsychopharmacol. 2021;24:392-399. https://doi.org/10.1093/ijnp/pyab001 33406269 PMC8130199

[55] Strick CA, Li C, Scott L, et al Modulation of NMDA receptor function by inhibition of D-amino acid oxidase in rodent brain. Neuropharmacology. 2011;61:1001-1015. https://doi.org/10.1016/j.neuropharm.2011.06.029 21763704

[56] Hondo T, Warizaya M, Niimi T, et al 4-Hydroxypyridazin-3(2H)-one derivatives as novel D-amino acid oxidase inhibitors. J Med Chem. 2013;56:3582-3592. https://doi.org/10.1021/jm400095b 23566269

[57] Sparey T, Abeywickrema P, Almond S, et al The discovery of fused pyrrole carboxylic acids as novel, potent D-amino acid oxidase (DAO) inhibitors. Bioorg Med Chem Lett. 2008;18:3386-3391. https://doi.org/10.1016/j.bmcl.2008.04.020 18455394

[58] Terry-Lorenzo RT, Chun LE, Brown SP, et al Novel human D-amino acid oxidase inhibitors stabilize an active-site lid-open conformation. Biosci Rep. 2014;34:e00133. https://doi.org/10.1042/BSR20140071 25001371 PMC4127593

[59] Sacchi S, Rosini E, Pollegioni L, Molla G. D-amino acid oxidase inhibitors as a novel class of drugs for schizophrenia therapy. Curr Pharm Des. 2013;19:2499-2511. https://doi.org/10.2174/1381612811319140002 23116391

[60] Fradley R, Goetghebeur P, Miller D, et al Luvadaxistat: a novel potent and selective D-amino acid oxidase inhibitor improves cognitive and social deficits in rodent models for schizophrenia. Neurochem Res. 2023;48:3027-3041. https://doi.org/10.1007/s11064-023-03956-2 37289348 PMC10471729

[61] Fradley R, Goetghebeur P, Miller D, Burley R, Serrats J. Pre-clinical assessment of TAK-831, a selective D-amino acid oxidase inhibitor, in animal models of schizophrenia. Schizophr Bull. 2019;45:S313-S314. https://doi.org/10.1093/schbul/sbz020.567

[62] Serrats J, Fradley R, Murthy V, O’Donnell P, Asgharnejad M. Pre-clinical evaluation of hippocampal long-term potentiation after acute and sub-chronic oral dosing with luvadaxistat in mice. Neuropsychopharmacol. 2021;46:322-323.

[63] Okamura H, Yasugaki S, Suzuki-Abe H, et al Long-term effects of repeated social defeat stress on brain activity during social interaction in BALB/c mice. eNeuro. 2022;9:ENEURO.0068-ENEU22.2022. https://doi.org/10.1523/ENEURO.0068-22.2022 35437264 PMC9070729

[64] Bolbecker AR, Kent JS, Petersen IT, et al Impaired cerebellar-dependent eyeblink conditioning in first-degree relatives of individuals with schizophrenia. Schizophr Bull. 2014;40:1001-1010. https://doi.org/10.1093/schbul/sbt112 23962891 PMC4133656

[65] Reeb-Sutherland BC, Fox NA. Eyeblink conditioning: a non-invasive biomarker for neurodevelopmental disorders. J Autism Dev Disord. 2015;45:376-394. https://doi.org/10.1007/s10803-013-1905-9 23942847

[66] Volianskis A, France G, Jensen MS, et al Long-term potentiation and the role of N-methyl-D-aspartate receptors. Brain Res. 2015;1621:5-16. https://doi.org/10.1016/j.brainres.2015.01.016 25619552 PMC4563944

[67] Luscher C, Malenka RC. NMDA receptor-dependent long-term potentiation and long-term depression (LTP/LTD). Cold Spring Harb Perspect Biol. 2012;4:a005710. https://doi.org/10.1101/cshperspect.a005710 22510460 PMC3367554

[68] Yoneyama T, Sato S, Sykes A, et al Mechanistic multilayer quantitative model for nonlinear pharmacokinetics, target occupancy and pharmacodynamics (PK/TO/PD) relationship of D-amino acid oxidase inhibitor, TAK-831 in mice. Pharm Res. 2020;37:164. https://doi.org/10.1007/s11095-020-02893-x 32901384 PMC7478952

[69] Xu L, DeMartinis N, Wu J, et al Safety, pharmacokinetics, and pharmacodynamics of TAK-831, a selective D-amino acid oxidase inhibitor, in healthy volunteers. Abstract W187. Neuropsychopharmacol. 2018;43:S476-S477.

[70] Berkhout J, Post T, Xu L, et al Application of population pharmacokinetic/pharmacodynamic (PK/PD) modeling and simulation to inform the design of a dose-finding study in patients with schizophrenia. Poster presented during the annual meeting of the Population Approach Group in Europe (PAGE), June 11-14, 2019, Stockholm, Sweden. Accessed January 3, 2025. https://www.page-meeting.org/pdf_assets/5246-TAK-831_2019%20PAGE%20Poster%20Encore.pdf

[71] O’Donnell P, Dong C, Murthy V, et al The D-amino acid oxidase inhibitor luvadaxistat improves mismatch negativity in patients with schizophrenia in a randomized trial. Neuropsychopharmacology. 2023;48:1052-1059. https://doi.org/10.1038/s41386-023-01560-0 36928351 PMC10018616

[72] Javitt DC, Doneshka P, Zylberman I, Ritter W, Vaughan HG Jr. Impairment of early cortical processing in schizophrenia: an event-related potential confirmation study. Biol Psychiatry. 1993;33:513-519. https://doi.org/10.1016/0006-3223(93)90005-x 8513035

[73] Umbricht D, Krljes S. Mismatch negativity in schizophrenia: a meta-analysis. Schizophr Res. 2005;76:1-23. https://doi.org/10.1016/j.schres.2004.12.002 15927795

[74] Light GA, Hsu JL, Hsieh MH, et al Gamma band oscillations reveal neural network cortical coherence dysfunction in schizophrenia patients. Biol Psychiatry. 2006;60:1231-1240. https://doi.org/10.1016/j.biopsych.2006.03.055 16893524

[75] Murthy V, Hanson E, DeMartinis N, et al INTERACT: a randomized phase 2 study of the DAAO inhibitor luvadaxistat in adults with schizophrenia. Schizophr Res. 2024;270:249-257. https://doi.org/10.1016/j.schres.2024.06.017 38943928

[76] Fan R, Brar S, Singh J, et al Use of digital health technology tool to assess medication adherence in schizophrenia: the INTERACT study experience [poster]. Presented at the International Society for CNS Clinical TrialA and Methodology (ISCTM) Autumn Conference October 5-7 2023, Barcelona, Spain. 2023.

[77] Fan R , et al Evaluating the potential impact of digital health technology tool use on efficacy and adherence in schizophrenia trials [poster]. Presented at the International Society for CNS Clinical Trials and Methodology (ISCTM) 20th Annual Scientific Meeting, February 21-23, 2024, Washington DC, USA. 2024.

[78] Fan R , et al Incorporating digital health technology tools in study design to assess medication adherence in schizophrenia trials [poster]. Presented at the 2024 Annual Congress of the Schizophrenia International Research Society, April 3-7, 2024, Florence, Italy. 2024.

[79] Neurocrine Biosciences Press Release. Neurocrine biosciences provides update on ERUDITE™ Phase 2 data for luvadaxistat in adults with cognitive impairment associated with schizophrenia. 2024. Accessed September 17, 2024. https://neurocrine.gcs-web.com/news-releases/news-release-details/neurocrine-biosciences-provides-update-eruditetm-phase-2-data.

[80] Umbricht D, Alberati D, Martin-Facklam M, et al Effect of bitopertin, a glycine reuptake inhibitor, on negative symptoms of schizophrenia: a randomized, double-blind, proof-of-concept study. JAMA Psychiatry. 2014;71:637-646. https://doi.org/10.1001/jamapsychiatry.2014.163 24696094

[81] Bugarski-Kirola D, Blaettler T, Arango C, et al Bitopertin in negative symptoms of schizophrenia-results from the phase III FlashLyte and DayLyte studies. Biol Psychiatry. 2017;82:8-16. https://doi.org/10.1016/j.biopsych.2016.11.014 28117049

[82] Fleischhacker WW, Podhorna J, Gröschl M, et al Efficacy and safety of the novel glycine transporter inhibitor BI 425809 once daily in patients with schizophrenia: a double-blind, randomised, placebo-controlled phase 2 study. Lancet Psychiatry 2021;8:191-201. https://doi.org/10.1016/S2215-0366(20)30513-7 33610228

[83] Fitapelli B, Lindenmayer JP. Advances in cognitive remediation training in schizophrenia: a review. Brain Sci 2022;12:129. https://doi.org/10.3390/brainsci12020129 35203893 PMC8870375

[84] Rosenbrock H, Desch M, Wunderlich G. Development of the novel GlyT1 inhibitor, iclepertin (BI 425809), for the treatment of cognitive impairment associated with schizophrenia. Eur Arch Psychiatry Clin Neurosci. 2023;273:1557-1566. https://doi.org/10.1007/s00406-023-01576-z 36971864 PMC10465677

[85] Wong DF, Ostrowitzki S, Zhou Y, et al Characterization of [11C]RO5013853, a novel PET tracer for the glycine transporter type 1 (GlyT1) in humans. Neuroimage. 2013;75:282-290. https://doi.org/10.1016/j.neuroimage.2011.11.052 22155032

[86] Buller AL, Larson HC, Schneider BE, et al The molecular basis of NMDA receptor subtypes: native receptor diversity is predicted by subunit composition. J Neurosci. 1994;14:5471-5484. https://doi.org/10.1523/JNEUROSCI.14-09-05471.1994 7916045 PMC6577092

[87] Laurie DJ, Seeburg PH. Regional and developmental heterogeneity in splicing of the rat brain NMDAR1 mRNA. J Neurosci. 1994;14:3180-3194. https://doi.org/10.1523/JNEUROSCI.14-05-03180.1994 8182465 PMC6577450

[88] Priestley T, Laughton P, Myers J, et al Pharmacological properties of recombinant human N-methyl-D-aspartate receptors comprising NR1a/NR2A and NR1a/NR2B subunit assemblies expressed in permanently transfected mouse fibroblast cells. Mol Pharmacol. 1995;48:841-848. 7476914

[89] Matsui T, Sekiguchi M, Hashimoto A, et al Functional comparison of D-serine and glycine in rodents: the effect on cloned NMDA receptors and the extracellular concentration. J Neurochem. 1995;65:454-458. https://doi.org/10.1046/j.1471-4159.1995.65010454.x 7790891

[90] de Bartolomeis A, Vellucci L, Austin MC, De Simone G, Barone A. Rational and translational implications of D-amino acids for treatment-resistant schizophrenia: from neurobiology to the clinics. Biomolecules. 2022;12:909. 35883465 10.3390/biom12070909 PMC9312470

[91] Hardingham GE, Bading H. Synaptic versus extrasynaptic NMDA receptor signalling: implications for neurodegenerative disorders. Nat Rev Neurosci. 2010;11:682-696. https://doi.org/10.1038/nrn2911 20842175 PMC2948541

[92] Papouin T, Ladépêche L, Ruel J, et al Synaptic and extrasynaptic NMDA receptors are gated by different endogenous coagonists. Cell. 2012;150:633-646. https://doi.org/10.1016/j.cell.2012.06.029 22863013

[93] Li Y, Sacchi S, Pollegioni L, et al Identity of endogenous NMDAR glycine site agonist in amygdala is determined by synaptic activity level. Nat Commun. 2013;4:1760. https://doi.org/10.1038/ncomms2779 23612301 PMC3641574

[94] Billups D, Attwell D. Active release of glycine or D-serine saturates the glycine site of NMDA receptors at the cerebellar mossy fibre to granule cell synapse. Eur J Neurosci. 2003;18:2975-2980. https://doi.org/10.1111/j.1460-9568.2003.02996.x 14656293

[95] Nong Y, Huang Y-Q, Ju W, et al Glycine binding primes NMDA receptor internalization. Nature. 2003;422:302-307. https://doi.org/10.1038/nature01497 12646920

[96] Martina M, Gorfinkel Y, Halman S, et al Glycine transporter type 1 blockade changes NMDA receptor-mediated responses and LTP in hippocampal CA1 pyramidal cells by altering extracellular glycine levels. J Physiol. 2004;557:489-500. https://doi.org/10.1113/jphysiol.2004.063321 15064326 PMC1665089

[97] Lin CH, Chen PK, Wang SH, Lane HY. Effect of sodium benzoate on cognitive function among patients with behavioral and psychological symptoms of dementia: secondary analysis of a randomized clinical trial. JAMA Netw Open. 2021;4:e216156. https://doi.org/10.1001/jamanetworkopen.2021.6156 33881530 PMC8060832

[98] Lin CH, Yang HT, Chen PK, Wang SH, Lane HY. Precision medicine of sodium benzoate for the treatment of Behavioral and Psychological Symptoms of Dementia (BPSD). Neuropsychiatr Dis Treat. 2020;16:509-518. https://doi.org/10.2147/NDT.S234371 32110025 PMC7039065

[99] Lin CH, Wang SH, Lane HY. Effects of sodium benzoate, a D-Amino acid oxidase inhibitor, on perceived stress and cognitive function among patients with late-life depression: a randomized, double-blind, sertraline- and placebo-controlled trial. Int J Neuropsychopharmacol. 2022;25:545-555. https://doi.org/10.1093/ijnp/pyac006 35023557 PMC9352177

[100] Griffiths K, Smart SE, Barker GJ, et al Treatment resistance NMDA receptor pathway polygenic score is associated with brain glutamate in schizophrenia. Schizophr Res. 2023;260:152-159. https://doi.org/10.1016/j.schres.2023.08.020 37657282 PMC10873209

